# The impacts of tobacco and nicotine on HIV-1 infection, inflammation, and the blood-brain barrier in the central nervous system

**DOI:** 10.3389/fphar.2024.1477845

**Published:** 2024-10-28

**Authors:** Aislinn M. Keane, Talia H. Swartz

**Affiliations:** ^1^ Graduate School of Biomedical Sciences, Icahn School of Medicine at Mount Sinai, New York, NY, United States; ^2^ Division of Infectious Diseases, Department of Medicine, Icahn School of Medicine at Mount Sinai, New York, NY, United States

**Keywords:** HIV-1, NLRP3 inflammasome, neuroinflammation, nicotine, blood-brain barrier (BBB)

## Abstract

Human immunodeficiency virus (HIV-1) remains a persistent global health crisis. Even while successfully virologically suppressed, people with HIV (PWH) experience a higher risk for inflammatory disorders such as HIV-associated neurocognitive disorder (HAND). Tobacco use puts PWH at higher risk for neurocognitive symptoms resulting from HIV-associated neuroinflammation. The NLR Family Pyrin Domain Containing 3 (NLRP3) inflammasome has been implicated as a driver of HIV-associated inflammation, including HAND. Nicotine, the psychoactive component of tobacco smoke, has also been shown to signal through the NLRP3 inflammasome and modulate inflammatory signaling in the CNS. Here, we explore the impacts of nicotine and tobacco on the complex neurobiology of HAND, including effects on cognition, inflammation, viral latency, and blood-brain barrier integrity. We outline nicotine’s role in the establishment of active and latent infection in the brain and posit the NLRP3 inflammasome as a common pathway by which HIV-1 and nicotine promote neuroinflammation in PWH.

## Introduction

Human immunodeficiency virus (HIV-1) affects more than 39 million people worldwide and remains incurable despite major advances in antiretroviral therapy (ART). Although patient life expectancy and quality of life has dramatically improved over the past few decades, People with HIV (PWH) are still at greater risk for several comorbid conditions due to viral persistence in latent reservoirs and associated low-level chronic inflammation (Chun et al., 2010; Collora and Ho, 2022; Global, 2024; Lee et al., 2020; Siliciano et al., 2003; White et al., 2022). These include a variety of conditions related to aging, including cardiovascular disease, osteoporosis, cancer, and neurocognitive disorders (Aberg, 2012; Guaraldi et al., 2011; Heaton et al., 2010; Kaplan-Lewis et al., 2017; Kearns et al., 2017). PWH also tend to develop these conditions earlier than the general population (Aberg, 2012; Kaplan-Lewis et al., 2017). Chronic inflammation due to persistent infection in viral reservoirs such as the central nervous system (CNS) is thought to be the primary driver of this increased risk (Desplats et al., 2013; Sonti et al., 2021).

HIV-associated neurocognitive Disorder (HAND) is a common HIV-associated condition affecting 20%–50% of PWH (Heaton et al., 2010). HAND is characterized by increased neuroinflammation, blood-brain barrier (BBB) breakdown, metabolic dysfunction, and measurable cognitive impairment (Saylor et al., 2016). Drug use, including smoking, is a risk factor for the development of HAND in PWH (Saylor et al., 2016). Tobacco users with HIV experience poorer outcomes, including increased risk of mortality and virologic rebound and poorer response to cART (Han et al., 2018). Because of the high prevalence of tobacco use in PWH and its known detrimental effects, unraveling its role in neuroinflammation will provide much-needed insights into mechanisms and possible therapeutic strategies for PWH.

## Biology of HAND: Chronic inflammation in the CNS

People with HIV are more likely to exhibit markers of inflammation even when viral levels are suppressed below detection by long-term ART treatments (Aberg, 2012; Deeks, 2011; Sieg et al., 2021). In virologically suppressed individuals, replication persists in tissues where ART has limited penetrance (Kulpa and Chomont, 2024). Despite ART, this leads to persistent immune activation, senescence, and an increased systemic inflammatory milieu (Aberg, 2012; Deeks, 2011). The brain is a viral reservoir with unique characteristics due to its immune-privileged status and thus presents a distinct set of challenges for a sterilizing cure. Although improved access to ART has greatly improved the quality of life and life expectancy for PWH, even complete viral suppression under ART treatment does not eliminate the cognitive symptoms associated with HIV infection of the brain (Heaton et al., 2010). HAND is defined clinically as a spectrum of disorders ranging from Asymptomatic Neurocognitive Impairment (ANI) to Mild Neurocognitive Disorder (MND) to HIV-associated Dementia (HAD), the most severe presentation (Heaton et al., 2011). Clinical studies have shown that biomarkers associated with CNS inflammation in blood plasma and cerebrospinal fluid (CSF) are more prevalent during acute HIV infection and decline over time and with ART treatment (Longino et al., 2022). As access to earlier ART intervention improves, rates of the more severe forms of HAND have been declining. Still, it is estimated that between 20% and 50% of PWH experience some form of neurocognitive impairment due to chronic HIV infection (Heaton et al., 2010).

Neuroinflammation in the HIV-1 infected CNS is characterized by BBB dysfunction, immune cell infiltration, and infection and inflammatory signaling of resident CNS cells, particularly microglia (Khanal et al., 2021; Saylor et al., 2016; Sreeram et al., 2022). Infected microglia compose the bulk of the viral reservoir in the CNS, and HIV is seeded into the brain in the very early stages of infection, primarily by infected monocytes, which migrate across the blood-brain barrier (Davis et al., 1992; Kahn and Walker, 1998; Longino et al., 2022; Valcour et al., 2012). Damage to the blood-brain barrier is a key aspect of many neuroinflammatory conditions, and chronic neuroinflammation from HIV infection is no exception. BBB dysfunction persists even in the presence of ART and during chronic infection (Kulpa and Chomont, 2024). Brain microvascular endothelial cells (BMVECs), pericytes, and astrocytes, are all dysregulated by the presence of virus in the CNS (Andersson et al., 2001; Eugenin et al., 2011; Leibrand et al., 2017; Osborne et al., 2020; Piekna-Przybylska et al., 2019). Endothelial cells are prone to dysfunction and death due to direct interaction with viral proteins and exposure to inflammatory cytokines released by infected cells (Andersson et al., 2001; Lee et al., 2004; Leibrand et al., 2017). Dysregulation of endothelial tight junction proteins such as claudin-5, occludin, and ZO-1 leads to increased BBB permeability and may facilitate infiltration of peripheral immune cells to the CNS (Boven et al., 2000; Chaudhuri et al., 2008; Dallasta et al., 1999; Eugenin et al., 2011). Decreased pericytes coverage of the endothelium and infected or dysregulated astrocytes have also been shown to contribute to barrier dysfunction in the HIV-infected brain (Piekna-Przybylska et al., 2019; Pla-Tenorio et al., 2023; Valdebenito et al., 2021). Although HIV can both cross and dysregulate the BBB, BBB penetrance remains a complex problem for the efficacy of antiretroviral drugs. The viral reservoir in the brain is formed by a combination of this challenge for ART delivery and the development of a population of latently infected cells, primarily microglia, which harbor HIV provirus (Osborne et al., 2020).

As the cell population most susceptible to HIV infection in the brain, microglia form the bulk of the latent reservoir (Wallet et al., 2019). An established literature shows a relationship between HIV-1 proteins, neuronal death, and microglial activation. Transgenic mouse studies show that viral proteins such as Tat and gp120 are produced by infected cells and have neurotoxic properties (Leibrand et al., 2017). Additionally, infected human microglia become reactive and release pro-inflammatory cytokines such as TNF⍺, CCL2, IL-1β, IL-6, and CCL5 (Alvarez-Carbonell et al., 2019). Infected microglia are also prone to mitochondrial dysfunction and overproduction of ROS (Alvarez-Carbonell et al., 2019; Borrajo et al., 2021).

Recent models suggest that cyclical reactivation of latent provirus in microglia may cause the ongoing cascade of inflammation in HAND (Sreeram et al., 2022). The susceptibility of microglial cells to latent infection is thought to be tied to the activation state at the time of infection, with more quiescent cells favoring a latent status than reactive or activated cells (Sreeram et al., 2022; Wallet et al., 2019). Activation of the transcription factor NF-κB or IRF3 in response to an inflammatory stimulus can reactivate viral replication in a latently infected human microglial cell line (Alvarez-Carbonell et al., 2017). Thus, inflammatory stimuli such as damaged neurons or drug exposures can potentiate latency reversal and promote the chronic inflammation characteristic of HAND (Alvarez-Carbonell et al., 2019). We will further explore the role of NLRP3 inflammasome signaling and nicotine exposures in driving this complex inflammatory cascade.

## HIV and the NLRP inflamamsome

The NLR Family Pyrin Domain Containing 3 (NLRP3) inflammasome, which consists of NLRP3, ASC, and pro-caspase-1, has been implicated in HAND and other HIV-associated inflammatory pathologies. Upon activation by intracellular pathogens and binding of Damage-associated Molecular Patterns (DAMP) to PRRs (Pattern Recognition Receptors), the inflammasome activates caspase-1, leading to the release of pro-inflammatory cytokines (IL-1β and Il-18) and pyroptosis, an inflammatory form of cell death known to contribute to inflammation in PWH (Doitsh et al., 2014; Katuri et al., 2019; Mamik and Power, 2017). HIV infection in T-cells may lead to incomplete transcription and viral fragments, triggering PRRs and leading to NLRP3 activation, inflammatory cytokine release, and cell death (Doitsh et al., 2014). In myeloid cells, NLRP3 activation and inflammatory cytokine release are associated with many HIV-associated inflammatory processes, such as atherosclerosis (Mao et al., 2021; Mullis and Swartz, 2020).

Animal studies in the CNS have shown that the NLRP3 inflammasome can be activated in microglia in response to viral proteins such as Tat, Vpr, and gp120, resulting in inflammatory cytokine release and neuroinflammation (Chivero et al., 2017; He et al., 2020). HIV infection was shown to promote IL-1β release in primary human microglia (Walsh et al., 2014). Furthermore, NLRP3 inflammasome inhibitors have shown a therapeutic effect in a mouse HAND model (He et al., 2020; Mamik and Power, 2017).

Alongside microglia-induced inflammatory signaling, the NLRP3 inflammasome is also implicated in BBB dysregulation. HIV-1 dysregulates the BBB through a variety of mechanisms, from direct interaction of brain microvascular endothelial cells (BMVECs) with viral proteins to inflammatory cytokine release (Il-6, TNF-a, IL-1β) from infected microglia and infiltrating monocytes in the CNS (Atluri et al., 2015; Caligaris et al., 2021). Studies indicate that HIV readily establishes latency in microglia and that microglial activation by NLRP3-associated inflammatory cytokines such as TNF-α and IL-1β may contribute to viral emergence from proviral latency, contributing to the chronic inflammation of HAND (Alvarez-Carbonell et al., 2017; Alvarez-Carbonell et al., 2019; Ko et al., 2019; Li and Barres, 2018; Plaza-Jennings et al., 2022; Sreeram et al., 2022).

## Nicotine and inflammation

Nicotine and its effects in the context of HAND are particularly of interest due to the high prevalence of smoking among PWH and the heightened health risks experienced by PWH who smoke. The established literature points to tobacco use as contributing to inflammation in a variety of contexts, both inside and outside of the CNS. However, its pro-inflammatory and anti-inflammatory effects can be disease and context dependent. Nicotine signals through the Nicotinic Acetylcholine receptor family (nAChRs), which are involved in cholinergic signaling and reward stimulus (de Kloet et al., 2015). They are also widely expressed in various cell types, including monocytes, macrophages, and microglia (Richter and Grau, 2023; Suzuki et al., 2006; Zoli et al., 2018). In non-excitable cells, signaling is primarily mediated through the influx of calcium ions directly through the ion-gated channel of the receptor (Zoli et al., 2018). In different non-neuronal cell types, signaling can be ionically or non-ionically driven and trigger downstream signaling via various pathways (Sorimachi et al., 1994; Zia et al., 2000; Zoli et al., 2018).

The general pro- and anti-inflammatory properties of nicotine are complex and context-dependent, with factors such as cell type, receptor type, and disease context playing important roles. In many studies, nicotine exhibits anti-inflammatory properties due to cholinergic signaling in neurons and non-neuronal cells (Zoli et al., 2018). However, the literature consistently links nicotine and NLRP3 inflammasome activation, especially in myeloid-derived cell types. There is a well-established relationship between nicotine and NLRP3-driven atherosclerosis progression (Duan et al., 2021; Mullis and Swartz, 2020; Wu et al., 2018; Xu et al., 2021). One study demonstrated that nicotine promotes NLRP3 inflammasome activation in peripheral myeloid cells, driving atherosclerosis progression due to inflammatory cytokine release in macrophages and monocytes (Mao et al., 2021). Nicotine was also shown to exacerbate proliferation and cell migration in lung adenocarcinoma via a5-nAChR and NLRP3 signaling (Jia et al., 2022).

In the context of the CNS, nicotine exerts direct pro-inflammatory effects on both microglia and the vasculature of the BBB. Exposure to NNKs (nicotine-derived nitrosamine ketones, a nicotine-derived metabolite) increased ROS and inflammatory cytokine release in mouse microglia (Ghosh et al., 2009). Tobacco use is generally associated with BBB disruption, CNS oxidative stress, and decreased cognitive performance in clinical studies (Ande et al., 2013; Ghosh et al., 2009; Louboutin and Strayer, 2014; Mazzone et al., 2010). BBB damage due to nicotine exposure is primarily linked to mitochondrial dysfunction, ROS production, and release of inflammatory cytokines such as TNFa and IL-6, which can trigger endothelial dysfunction by dysregulation of tight junction proteins such as claudin-5 and occludin (Hossain et al., 2011; Hutamekalin et al., 2008; Kousik et al., 2012; Manda, Mittapalli, Geldenhuys, et al., 2010; Pimentel et al., 2020). Additionally, Zhang et al. have linked the NLRP3 inflammasome directly to nicotine-induced endothelial barrier dysfunction and hyperpermeability via the release of HMGB1 (Zhang et al., 2019).

## HIV and nicotine in HAND

While nicotine and HIV alone both contribute to neuroinflammation and BBB dysfunction, in PWH who smoke, additive effects have been observed on neuroinflammation and decreased cognitive performance (Bryant et al., 2013; Chang et al., 2020). These clinical findings are supported by studies in HIV-1 transgenic rat models, where nicotine promotes overexpression of immune-related genes and inflammatory cytokine expression (Royal et al., 2018; Yang et al., 2016). Additionally, Delgado-Velez et al. showed that gp120 exposure can directly upregulate the expression of a7-nAChRs in peripheral immune cells (Delgado-Vélez et al., 2015). In addition to promoting their upregulation, the viral protein gp120 can bind to the α7 receptors. α7-nAChR signaling promoted amyloid-beta accumulation in an HIV-gp120 mouse model (Liu et al., 2017). While certain animal studies show that nicotine alone increases cognitive performance in behavioral tests, this improvement is ameliorated in HIV-1 Tg rats (Nizri et al., 2009; Revathikumar et al., 2016; Royal et al., 2018; Yang et al., 2016). Along a similar vein, α7-nAChRs were found to be upregulated in the monocytes of women with HIV, but greater receptor abundance did not result in a protective anti-inflammatory effect when MDMs were treated with LPS (Delgado-Vélez et al., 2015). These findings, when taken together, suggest that the presence of HIV may alter the cholinergic anti-inflammatory response via the α7-nAChRs, dampening or even reversing the anti-inflammatory effects of nicotine alone.

It is important to consider that in people who smoke cigarettes, nicotine is always accompanied by the thousands of other compounds present in tobacco smoke. Tobacco smoke and nicotine alone have been shown to have differing effects on inflammation, and their respective contributions to HIV associated neuroinflammation are not yet clearly delineated. Tobacco smoke is almost universally pro-inflammatory in both pre-clinical and clinical studies. Cigarette smoke condensate increases apoptosis, viral replication, and oxidative stress in human monocytes (Rao et al., 2016). Tobacco smoke extract similarly increases viral replication in bronchial epithelial cells and alveolar macrophages (Abbud et al., 1995; Chinnapaiyan et al., 2018). In clinical studies, PWH who smoke exhibit higher levels of inflammatory markers, increased viral load, and increased risk of HAND compared to non-smokers (Han et al., 2018; Valiathan et al., 2014; Wojna et al., 2007). As mentioned above, nicotine can induce anti-inflammatory cholinergic signaling through the ⍺7 nAChR and can be neuroprotective in certain contexts, including in some HIV-1 Tg rat studies (Cao et al., 2013; Cao et al., 2016). However, chronic nicotine exposure is known to cause BBB disruption, a key component of HAND pathology (Feldman and Anderson, 2013). Furthermore, there is evidence that nicotine treatment promotes HIV infection in human microglia (Rock et al., 2008). Further studies with both nicotine and tobacco smoke are needed to unravel their roles in the context of HAND.

In addition to the many compounds present in tobacco smoke, antiretroviral drug treatments and other substance use in PWH add another layer of complexity to the neuroinflammatory context of the HIV-infected CNS. Many studies have shown that earlier intervention with ART can significantly reduce the risk of more severe forms of HAND in PWH (Brew, 2004; Sacktor et al., 2002). However, as outlined earlier, infection and inflammation persist in the brain despite the presence of ART. As reviewed elsewhere, certain antiretroviral drugs can have neurotoxic effects through mechanisms such as oxidative stress and mitochondrial dysfunction, and balancing this neurotoxicity against viral suppression and penetrance of the CNS reservoir remains an important clinical challenge (Shah et al., 2016; Yuan and Kaul, 2021). In PWH who smoke tobacco, there is the additional factor of drug-drug interactions between nicotine and ARTs (reviewed by Ghura et al., 2020). Nicotine has been shown to affect ART metabolism directly and can impact drug delivery by compromising BBB integrity (Kumar et al., 2015; Manda, Mittapalli, Bohn, et al., 2010; Manda, Mittapalli, Geldenhuys, et al., 2010; Pal et al., 2011). Animal model studies with the protease inhibitor saquinavir demonstrate that nicotine-induced BBB compromise may facilitate entry of ART into the CNS, but nicotine and saquinavir cause additive oxidative stress in the brain endothelium leading to dysregulation of Notch-4 and ZO-1 (Manda, Mittapalli, Bohn, et al., 2010; Manda, Mittapalli, Geldenhuys, et al., 2010). Balancing ART toxicity with BBB penetrance remains an important clinical problem, as long-term exposure to certain ARTs has been associated with neurovascular toxicity (Bertrand et al., 2021). Thus, although nicotine’s ability to disrupt the BBB may lead to greater drug penetrance, it is likely that the additive impacts on BBB dysfunction and subsequent inflammation and immune infiltration of the CNS represent an overall detrimental outcome (Ahmed et al., 2018; Bertrand et al., 2021). ART has also been observed to increase nicotine metabolism in PWH (Ashare et al., 2019; Earla et al., 2014). ARTs may also impact nicotine signaling by acting on nicotinic receptors. For example, the protease inhibitor indinavir was demonstrated to impact cholinergic signaling by inhibiting the α7-nAChR activity (Ekins et al., 2017). More investigation is necessary to understand the specific interactions between various ARTs and nicotine to inform best practices for the treatment of PWH.

As outlined here, the NLRP3 inflammasome pathway is a common mechanism by which HIV and nicotine promote neuroinflammation. More studies are needed to fully understand how HIV and nicotine interact to promote inflammation, BBB dysregulation, and viral reactivation in the CNS.

## Discussion

Chronic inflammation leads to increased risks for comorbid disorders in PWH, even in the presence of ART (Chun et al., 2010; Collora and Ho, 2022; Lee et al., 2020; Siliciano et al., 2003; White et al., 2022). In the brain, this manifests as an increased risk for neuroinflammation and cognitive impairment classified under the family of neurocognitive disorders known as HAND (Heaton et al., 2011; Saylor et al., 2016). The persistence of HIV in viral reservoirs such as the CNS is a primary driver of this chronic inflammation (Desplats et al., 2013; Sonti et al., 2021). The use of substances such as tobacco increases the risk of comorbidities for PWH and further contributes to HIV-associated inflammatory pathologies (Han et al., 2018).

There is a well-established literature linking HIV-1 with NLRP3 inflammasome signaling both in the periphery and in the CNS. Studies have shown that HIV can stimulate the inflammasome through the binding of viral fragments and proteins to PRRs and through the activation of purinergic receptors (Doitsh et al., 2014; Freeman and Swartz, 2020; Swartz et al., 2015). In the brain, HIV drives NLRP3-associated inflammation primarily through infected and activated microglia, which contribute to the inflammatory environment by releasing neurotoxic factors and pro-inflammatory cytokines (Chivero et al., 2017; He et al., 2020; Walsh et al., 2014). HIV also drives BBB damage through several mechanisms, including NLRP3-driven dysregulation of endothelial cells (Atluri et al., 2015; Caligaris et al., 2021).

NLRP3 inflammasome activation, a key feature of neuroinflammation and BBB dysregulation, is influenced by both HIV and nicotine. Like HIV infection, nicotine is known to drive endothelial dysfunction by promoting ROS, mitochondrial dysfunction, and NLRP3 inflammasome activation (Ghosh et al., 2009; Hossain et al., 2011; Hutamekalin et al., 2008; Kousik et al., 2012; Manda, Mittapalli, Bohn, et al., 2010; Pimentel et al., 2020; Zhang et al., 2019). There is evidence that nicotine in tobacco enhances viral replication in microglia and macrophages and has been shown to cause activation of these resident immune cells and compromise the BBB (Ghosh et al., 2009; Manda, Mittapalli, Geldenhuys, et al., 2010; Pimentel et al., 2020; Rock et al., 2008). Taken together, the literature suggests that the combined effect of HIV and nicotine-driven NLRP3 activation plays a key role in the heightened neuroinflammation and cognitive symptoms observed in PWH who smoke.

Further investigation is necessary to understand the mechanism of HIV and nicotine’s interactions in the CNS and the precise role of NLRP3 in driving their combined effects. Furthermore, there are many critical unanswered questions surrounding the impact of substance use on the establishment and reactivation of viral latency in the CNS. Atluri et al. suggest that nicotine increases the risk of viral latency establishment due to the upregulation of HDAC2 in a neuronal cell line (Atluri et al., 2014). In this model, the transcriptional repressor HDAC2 is synergistically upregulated by HIV and nicotine, leading to more compact chromatin organization, reduced gene transcription, and increased latent infection. Treatment with the HDAC inhibitor vorinostat reversed this effect and reactivated latent virus. However, is important to note that the biological relevance of this study is limited, as neurons are not typically infected with HIV-1 and do not form a significant portion of the CNS viral reservoir. In a murine macrophage model of atherosclerosis, another histone deacetylase, HDAC6, was shown to promote nicotine-mediated inflammation and pyroptosis via deacetylation of p65, and activation of NF-kB and NLRP3 transcription (Xu et al., 2021). HIV-1 is known to integrate itself into transcriptionally active regions of the genome located in regions of open chromatin (Schröder et al., 2002). It relies on host cell machinery and activation states to regulate its latency, favoring activated over quiescent states in both microglia and CD4^+^ T-cells (Mbonye and Karn, 2017; Sreeram et al., 2022; Wallet et al., 2019). All of this considered, it seems likely that nicotine may play a role in regulating viral latency in microglia by promoting activation of NF-kB and NLRP3 associated genes, leading to transcription of latent provirus. However, nicotine’s specific role in viral latency formation and maintenance in the CNS remains largely unexplored in the literature, and thorough investigation of its impacts on latent infection in microglia is especially necessary.

There are additionally many open questions regarding poly-substance use in PWH and the impacts of combined drug use on cognitive impairment in these patients. As previously reviewed by our group, cannabis use is largely associated with protective effects against inflammation in PWH, and CB2R signaling has been linked to reduced HIV-1 infection and NLRP3 inflammasome activation (Min et al., 2023). Further study is needed to better understand the interactions between the cannabinoid system, nicotine, and NLRP3 and the impacts of multiple drug exposures on neuroinflammation in PWH. Investigating these questions will provide valuable insights into the mechanism of nicotine’s impact on the pathogenesis of HIV-1-associated neurodegeneration, informing possibilities for future therapeutic development.

## Abbreviations

PWH, People with HIV; BBB, Blood brain barrier; nAChR, nicotinic acetylcholine receptor; HAND, HIV-associated neurocognitive disorder; ART, antiretroviral therapy.
